# Early Mobilization Within 48 Hours as a Determinant of Functional Independence After Hip Fracture Surgery: Evidence From a Cohort Study

**DOI:** 10.7759/cureus.115043

**Published:** 2026-08-23

**Authors:** Syed Taqi Askari Shah, Abdul Rahman Ali, Qossay Alsaafin, Ali Adeeb Ilyas, Nidhi Reji, Nadia A Zeidan, Nawras Alkhateeb, Mohamed Elemam, Yazan Wardeh, Abdulla Nidal, Yasir A Al-Humairi

**Affiliations:** 1 Trauma and Orthopaedics, Queen's Medical Centre, Nottingham University Hospitals NHS Trust, Nottingham, GBR; 2 General Surgery, Kettering General Hospital, Kettering, GBR; 3 Medicine, Royal Albert Edward Infirmary, Wigan, GBR; 4 Trauma and Orthopaedics, Wirral University Teaching Hospital NHS Foundation Trust, Birkenhead, GBR; 5 General Surgery, Rotherham General Hospital, The Rotherham NHS Foundation Trust, Rotherham, GBR; 6 Medicine and Surgery, The Hashemite University, Zarqa, JOR; 7 General Medicine, Azerbaijan Medical University, Baku, AZE; 8 Trauma Surgery, Dubai Health, Dubai, ARE; 9 Orthopaedics and Trauma Surgery, Mohammed Bin Rashid University of Medicine and Health Sciences, Dubai Health, Dubai, ARE; 10 Surgery, Dubai Health Authority, Dubai, ARE; 11 Orthopaedics and Trauma, Rashid Hospital, Dubai, ARE

**Keywords:** delayed mobilization, early mobilization, elderly, hip fracture, modified barthel index, new mobility score, rehabilitation

## Abstract

Background

Postoperative mobilization is an important aspect of rehabilitation after hip fracture surgery among older adults. However, the optimal timing of mobilization remains disputed. This study compared early and delayed postoperative mobilization and their associations with short-term functional outcomes.

Methods

We conducted an ambispective observational cohort study of 405 older patients (aged 60 years or older) who had undergone hip fracture surgery. Participants were divided into two groups based on the timing of their first assisted mobilization: early mobilization (≤48 hours) and delayed mobilization (>48 hours). The New Mobility Score (NMS) was used to assess functional mobility at baseline (based on pre-fracture recall) and four weeks postoperatively. At four weeks, functional independence was assessed using the Barthel Index (BI). We used the Mann-Whitney U test to compare the groups and ordinal logistic regression to assess predictors of functional independence at four weeks.

Results

Early mobilization was significantly associated with higher baseline and follow-up NMS scores and higher BI scores at four weeks (p < 0.001). Age and BMI were significantly negatively associated with BI scores (p < 0.001). Regression analysis showed that early mobilization, younger age, lower BMI, fewer comorbidities, better pre-fracture mobility, less dependence on assistive devices, type of surgery, and shorter time to surgery were independently associated with greater functional independence (p < 0.05). In contrast, gender was not significantly associated with functional outcomes.

Conclusion

Early mobilization within 48 hours after hip fracture surgery was strongly associated with greater mobility and functional independence at four weeks in older patients.

## Introduction

Hip fractures are considered among the most frequent and severe injuries in the elderly population and often result from low-energy falls associated with osteoporosis, impaired balance, or frailty [1,2]. These fractures are linked to high morbidity, mortality, and loss of independence; hence, their management is a significant public health issue [3]. Surgical intervention is the standard of care for restoring mobility and minimizing complications; however, postoperative recovery management also influences functional outcomes [4].

Postoperative mobilization is a key component of rehabilitation after hip fracture surgery, with the goal of preventing complications, including deep vein thrombosis, pulmonary infections, muscle atrophy, and pressure ulcers [5,6]. Early mobilization, typically within 24-48 hours after surgery, is widely recommended to enhance functional recovery, reduce hospital stay, and improve overall quality of life [7,8]. In contrast, delayed mobilization may be necessary in cases of medical instability, pain, or surgical complications but has been linked to prolonged immobility, an increased risk of postoperative events, and delayed restoration of pre-fracture functional capacity [9,10].

Hip fractures are a major global health concern among older adults, with rates rising as a result of increasing life expectancy and the aging population worldwide. These injuries are linked to high morbidity, mortality, and long-term disability, placing a heavy burden on healthcare systems [11,12]. Postoperative rehabilitation is an important factor in improving recovery outcomes, and the timing of mobilization should be given particular consideration [7]. Early mobilization is an important element of effective recovery regimens because it can reduce complications such as thromboembolism and muscle atrophy, as well as the length of hospital stay [13]. Nevertheless, the optimal timing of mobilization is difficult to establish in clinical practice because patient-specific factors, including frailty, comorbidities, and baseline functional status, introduce substantial variability, and the existing evidence provides limited guidance on how mobilization strategies should be tailored to the individual.

Study gap

Although hip fracture surgery is routinely performed in older adults, the timing of postoperative mobilization remains highly debatable. Although early mobilization is widely recommended, evidence comparing its association with short-term functional recovery with that of delayed mobilization in elderly patients remains limited. Delayed mobilization, conversely, may be necessary when immediate postoperative risks are present but can result in prolonged immobility and delayed rehabilitation. The lack of consensus contributes to variability in postoperative rehabilitation practices, and it remains unclear which approach yields better functional outcomes in elderly patients.

The impact of early versus delayed mobilization on recovery, functional ability, and independence in the days and weeks after surgery is also not well understood. These differences are worth exploring to identify methods for improving rehabilitation and mitigating the risks of prolonged immobility among older adults.

Objectives

The primary objective of this ambispective observational cohort study was to examine the association of early (≤48 hours) and delayed (>48 hours) postoperative mobilization with four-week functional outcomes among elderly patients undergoing hip fracture surgery. The primary functional outcome was functional independence at four weeks, assessed using the Barthel Index (BI), while postoperative mobility, assessed using the four-week New Mobility Score (NMS), was evaluated as an additional outcome. The analysis considered baseline pre-fracture mobility and clinically relevant fracture- and treatment-related factors.

## Materials and methods

Study design and setting

This ambispective observational cohort study was conducted at WATIM Medical and Dental Hospital, Rawat, Pakistan, to examine the association of early and delayed postoperative mobilization with functional recovery in elderly patients undergoing hip fracture surgery. The study included 405 patients admitted to orthopedic wards and postoperative units. The sample included participants with diverse demographic and clinical characteristics, including age, gender, and comorbidities. The decision regarding the type of surgery was based on the surgeon’s judgment and hospital policies. The study had an ambispective design, with demographic, surgical, and perioperative information retrospectively extracted from hospital records, while four-week postoperative functional outcomes were prospectively assessed after participant enrollment and the provision of informed consent.

Sample size and technique

The sample size was estimated using the following formula for observational studies:

 \[n = \frac{Z^2 \cdot p (1 - p)}{d^2}\]

Here, n represents the required sample size, Z = 1.96 at a 95% confidence level, p = 0.5 as the assumed prevalence, and d = 0.05 as the margin of error [14]. The calculated minimum sample size was 384; however, 405 patients were included to increase the statistical power of the study.

Convenience sampling was used, whereby eligible patients were approached during their hospital stay, informed about the study, and asked to provide written informed consent before participating.

Participant selection criteria

The inclusion criteria were patients aged 60 years or older with a confirmed diagnosis of hip fracture who were undergoing surgical repair and were able to participate in postoperative assessments. Cognitive deficits, severe neurological conditions, or other medical conditions that could interfere with mobility or the evaluation of functional independence were exclusion criteria. Cognitive impairment was assessed through routine clinical assessment at admission rather than using a formal standardized screening instrument. Baseline interviews were conducted only when patients were clinically alert and able to provide reliable responses. Patients exhibiting acute confusion or delirium or who were unable to provide reliable information were excluded. Because a standardized cognitive screening instrument was not used, the possibility of subjective classification and residual misclassification cannot be excluded.

A total of 450 patients were assessed for eligibility. Of these, 45 patients were excluded because of cognitive impairment (n = 20), refusal to provide informed consent (n = 15), or incomplete clinical data (n = 10). The remaining 405 participants were included in the final analysis and categorized into the early (n = 230) and delayed (n = 175) mobilization groups. Bilateral fractures were recorded as a separate clinical characteristic. The 10 participants with bilateral hip fractures were retained in the primary analysis because they met the study eligibility criteria; however, their bilateral status was considered a potential source of variation in mobility and rehabilitation outcomes. Available records did not consistently distinguish simultaneous bilateral fractures from a previous contralateral hip fracture; therefore, this distinction could not be reliably evaluated in the present analysis.

**Figure 1:**
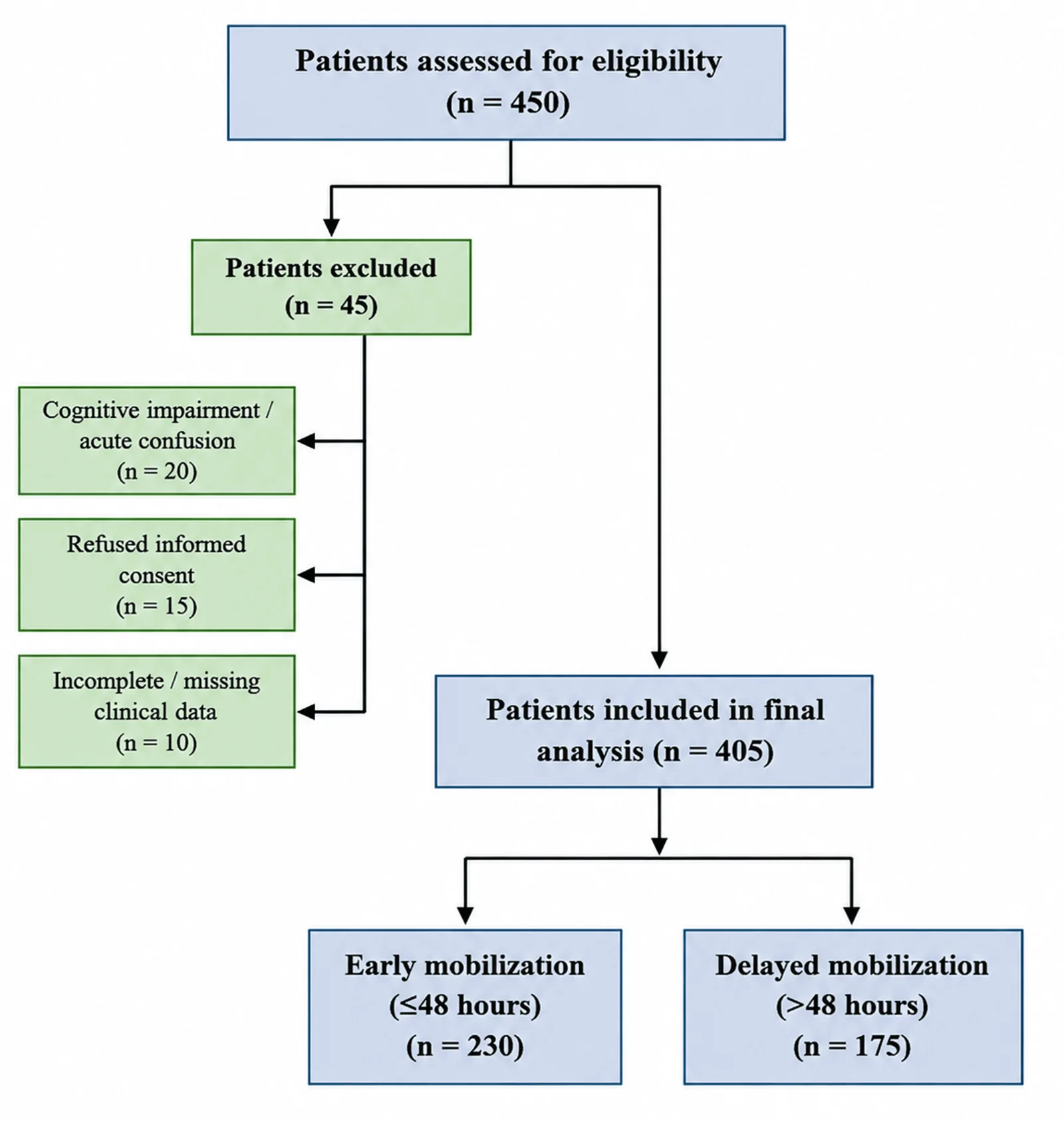
Flow diagram of participant selection for the study.

Figure 1 shows the flow diagram of participant recruitment, exclusions, and classification into the early (≤48 hours) and delayed (>48 hours) mobilization groups.

Research tools and measures

Demographic and Clinical Data

Background data were collected using a structured demographic form that included age, gender, comorbidities (e.g., hypertension, diabetes, and cardiovascular disease), type of hip fracture, type of surgery, and perioperative complications. A fracture-type-by-surgical-procedure cross-tabulation was not available in the retrospectively extracted clinical dataset, limiting the detailed assessment of procedure-specific treatment patterns. These data were critical for describing the study population, identifying possible confounding variables, and establishing subgroup analyses based on pertinent clinical factors.

Timing of Mobilization

The participants were divided into two groups based on the timing of their first assisted mobilization after hip fracture surgery. Patients who underwent their initial assisted mobilization within 48 hours after surgery were included in the early mobilization group. Assisted mobilization was defined as sitting at the edge of the bed, transferring to a chair, standing, or taking the first steps with support. Delayed mobilization was defined as initial assisted mobilization occurring more than 48 hours after surgery [15]. Nursing and physiotherapy records were reviewed to document the timing of mobilization accurately. This classification enabled functional recovery to be compared between the early and delayed mobilization groups.

Mobility Assessment

Functional mobility was evaluated using the NMS, developed by Parker MJ and Palmer CR in 1993. The NMS is a validated instrument that assesses a patient’s independence in performing basic mobility tasks. It comprises three items: indoor walking, outdoor walking, and shopping. Each item is rated on a scale from 0 to 3, giving a total score ranging from 0 to 9, with higher scores indicating greater mobility. The NMS is widely used and validated in hip fracture populations for assessing functional mobility and predicting postoperative outcomes [16].

The NMS was administered twice in this study: at baseline, through patient interviews, to assess recalled pre-fracture functional mobility, and at four weeks postoperatively, to determine recovery and mobility gains following early or delayed mobilization. To minimize recall bias, baseline pre-fracture mobility was assessed as early as possible after admission, and whenever feasible, responses were confirmed by accompanying family members or caregivers. Interviews were conducted only when patients were clinically able to provide reliable responses; patients with cognitive impairment or an inability to provide reliable information were excluded. The use of the NMS at two time points enabled the researchers to compare pre-injury and postoperative mobility and evaluate the association between mobilization timing and functional mobility recovery.

A validated ambulatory classification such as the Koval grade was not collected; therefore, pre-fracture ambulatory status was characterized using the NMS and predefined mobility categories. Specific postoperative weight-bearing and rehabilitation protocols were determined by the treating orthopedic team according to fracture characteristics, surgical procedure, fixation stability, and individual clinical status. These protocols were not standardized across all participants and were therefore not independently quantified for analysis. Mobilization timing was determined by the treating orthopedic team based on postoperative clinical stability and functional condition. Patients were mobilized after 48 hours when they were not considered clinically or functionally suitable for mobilization within the first 48 hours after surgery.

Functional Independence

Functional independence was evaluated using the BI, one of the most commonly used indices for assessing performance in activities of daily living (ADLs), developed by Mahoney FI and Barthel D in 1965. The BI measures the ability to perform 10 basic ADLs: feeding, bathing, grooming, dressing, bowel and bladder control, toilet use, transfers, mobility, and stair climbing. Items are rated according to the amount of assistance required, with higher scores indicating greater independence. The overall score ranges from 0 to 100, with higher scores indicating better functional status and greater independence in ADLs [17]. The original 10-item BI described by Mahoney FI and Barthel D was used in this study.

In the present study, the BI was administered once, four weeks after surgery, to assess the patient’s ability to perform ADLs following hip fracture surgery. It was selected as a measure of functional outcomes following postoperative mobilization because it reflects actual independence and recovery in everyday activities, which are important for elderly patients returning home or to community settings.

Procedure

The research was conducted from December 2024 to December 2025 in the orthopedic wards and postoperative units of WATIM Medical and Dental Hospital. Hospital records were used to identify eligible patients who had undergone hip fracture surgery. They were approached, informed about the study, and asked to provide written informed consent. Analgesic regimens and regional nerve blocks were not systematically collected because these interventions were not standardized across patients and were not consistently documented in a format suitable for uniform extraction during the study period. The intensity and frequency of physiotherapy and rehabilitation sessions were also not systematically quantified because rehabilitation schedules were individualized according to clinical status and were not recorded using a standardized protocol.

Participants were grouped into early (≤48 hours) and delayed (>48 hours) mobilization groups according to the time at which they first underwent assisted mobilization. Although the surgical procedure and postoperative restrictions were considered in clinical decision-making, mobilization timing was not determined solely by the type of surgery. The treating orthopedic team based mobilization primarily on overall postoperative clinical stability and the patient’s functional condition. Assisted mobilization was defined as the patient’s first supervised postoperative activity, including sitting at the edge of the bed, transferring to a chair, standing with assistance, or ambulating with an appropriate walking aid according to the patient’s pre-fracture functional status and postoperative clinical condition.

The NMS was used to assess functional mobility at baseline (based on pre-fracture recall) and four weeks postoperatively, and the BI was used to measure functional independence at four weeks postoperatively. The four-week follow-up assessment was performed during scheduled outpatient visits whenever feasible. Participants who were unable to attend the follow-up visit were contacted by telephone, and the BI and NMS assessments were completed using the same standardized assessment protocol. All functional assessments were performed by trained members of the research team using standardized assessment procedures. Because of the observational nature of the study, outcome assessors were not blinded to the participants’ mobilization status. All assessments were conducted in a standardized manner, and patient safety, privacy, and confidentiality were maintained throughout the study.

**Figure 2:**
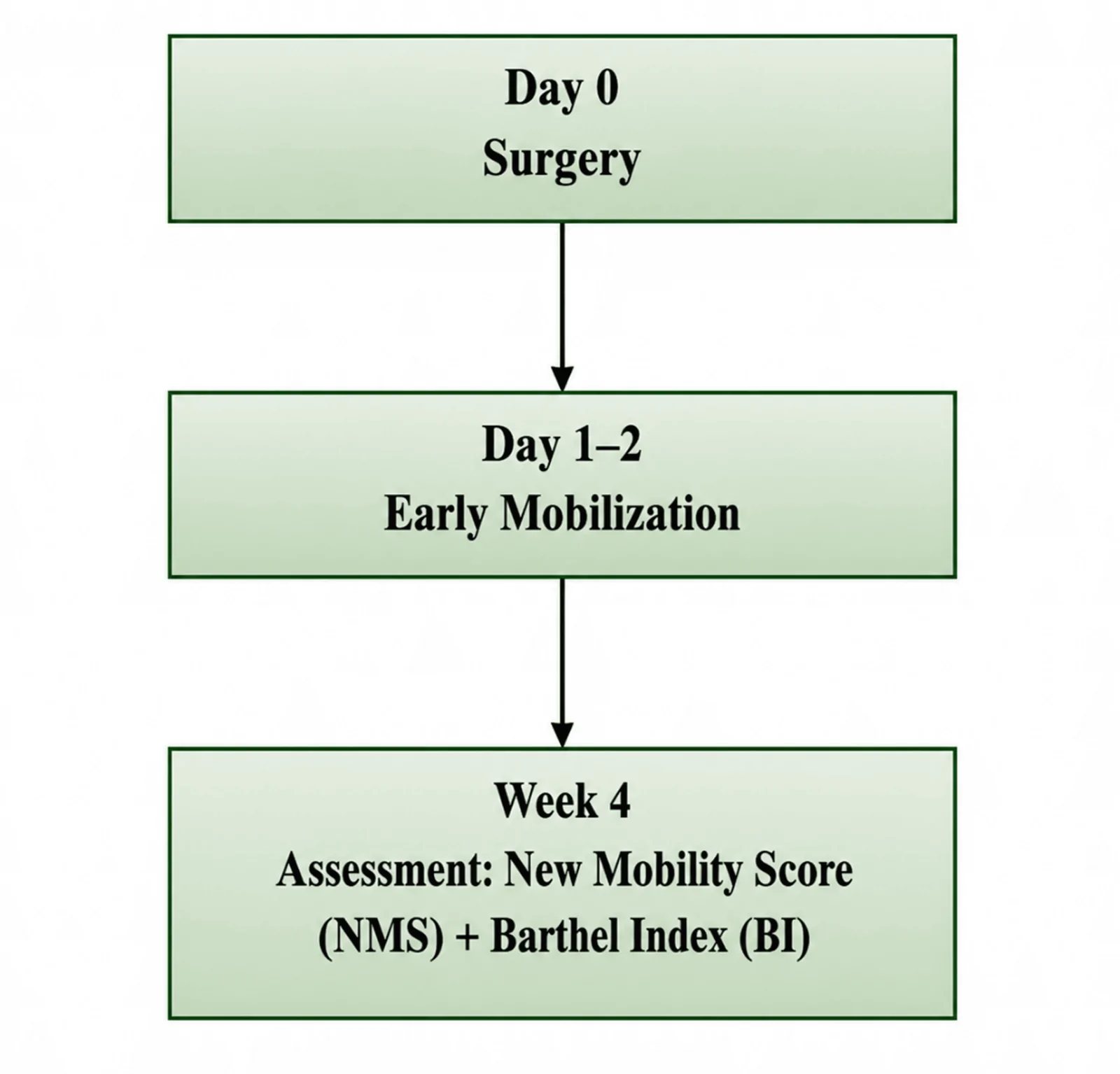
Clinical timeline of the study. Day 0: surgery; Days 1-2: early mobilization (≤48 hours); after Day 2: delayed mobilization (>48 hours); Week 4: assessment of functional outcomes using the New Mobility Score (NMS) and Barthel Index (BI).

Figure 2 shows the clinical timeline of the study, including surgery, early and delayed mobilization, and functional outcome assessment.

Analytical approach

Data were analyzed using IBM SPSS Statistics version 26.0 (IBM Corp., Armonk, NY, USA). Descriptive statistics were used to summarize demographic and clinical characteristics. Continuous variables were assessed for normality using the Shapiro-Wilk test. Because the study outcome variables were not normally distributed, comparisons between the early and delayed mobilization groups and between gender groups were performed using the Mann-Whitney U test. In contrast, comparisons across multiple age categories were conducted using the Kruskal-Wallis test. Spearman’s rank-order correlation was used to evaluate the relationships among age, body mass index, and BI scores.

Ordinal logistic regression analysis using a proportional-odds model was performed to identify independent predictors of the four-week functional independence category. Predictor variables included in the regression model were selected based on their clinical relevance and evidence from previous literature. The multivariable model included mobilization timing, age, gender, BMI, comorbidity burden, pre-fracture mobility, assistive device use, type of surgery, and time to surgery. Fracture-specific rehabilitation intensity, analgesic regimen, regional nerve blocks, pain severity, and postoperative weight-bearing restrictions were not systematically quantified and therefore could not be included as covariates. A two-sided p-value of <0.05 was considered statistically significant. Participants with incomplete clinical data were excluded before the analysis; therefore, no missing values were present in the final analytical dataset.

Ethical considerations

The study was approved by the Institutional Review Board of Watim Medical College (WMC/IRB/2024-780-orth). All participants provided written informed consent. Participation was voluntary, and patients were assured of confidentiality and the right to withdraw freely without affecting their treatment.

## Results

Table 1 presents the characteristics of the 405 participants. The largest age group was 71-75 years (n = 186, 45.9%), and the majority of participants were male (n = 246, 60.7%). Almost half were overweight (n = 195, 48.1%). The most frequent fracture type was an intertrochanteric fracture (n = 163, 40.2%), followed by a femoral neck fracture (n = 154, 38.0%), with a marginally higher number occurring on the right side (n = 207, 51.1%). The most common comorbidities were coronary artery disease (n = 122, 30.1%) and chronic kidney disease (n = 105, 25.9%). Before the fracture, almost half of the participants (n = 201, 49.6%) required a wheelchair or complete assistance, and 148 (36.5%) were nursing home residents. The most common surgical procedure was total hip replacement (n = 202, 49.9%), and 120 patients (29.6%) had a time to surgery of more than 72 hours.

**Table 1:** Demographic and clinical characteristics of participants (N = 405). N: Total sample size; n: Number of participants; %: Percentage; Pre-fracture mobility status: Baseline mobility status; COPD: Chronic obstructive pulmonary disease.

Variable	n	%
Age		
60-65 years	22	5.4
66-70 years	123	30.4
71-75 years	186	45.9
≥76 years	74	18.3
Gender		
Male	246	60.7
Female	159	39.3
BMI		
<18.5 (underweight)	8	2
18.5-24.9 (normal weight)	127	31.4
25.0-29.9 (overweight)	195	48.1
≥30.0 (obese)	75	18.5
Type of fracture		
Femoral neck fracture	154	38
Intertrochanteric fracture	163	40.2
Subtrochanteric fracture	88	21.7
Side of fracture		
Right	207	51.1
Left	188	46.4
Bilateral	10	2.5
Comorbidities		
Hypertension	23	5.7
Diabetes mellitus	87	21.5
Coronary artery disease	122	30.1
Chronic kidney disease	105	25.9
Osteoporosis	59	14.6
COPD/asthma	9	2.2
Pre-fracture mobility status		
Independent (walks without an aid)	94	23.2
Occasionally uses a cane or walker	110	27.2
Uses a wheelchair or requires full assistance	201	49.6
Living situation		
Alone	117	28.9
With spouse/family	140	34.6
Nursing home/assisted living	148	36.5
Smoking status		
Never smoked	138	34.1
Former smoker	198	48.9
Current smoker	69	17
Type of surgery		
Hemiarthroplasty	37	9.1
Total hip replacement	202	49.9
Internal fixation (pins/screws/plate)	166	41
Time from fracture to surgery (hours)		
<24 hours	81	20
24-48 hours	114	28.1
>48-72 hours	90	22.2
>72 hours	120	29.6
Assistive devices used pre-fracture		
None	63	15.6
Cane	134	33.1
Walker	94	23.2
Wheelchair	114	28.1

Table 2 shows significant differences between the early (n = 230) and delayed (n = 175) mobilization groups in age (p = 0.003), BMI (p = 0.041), diabetes (40 (17.4%) vs. 47 (26.9%), p = 0.026), coronary artery disease (58 (25.2%) vs. 64 (36.6%), p = 0.013), and chronic kidney disease (48 (20.9%) vs. 57 (32.6%), p = 0.008). Pre-fracture mobility (p < 0.001), living situation (p = 0.002), type of surgery (p = 0.021), time to surgery (p < 0.001), and assistive device use (p < 0.001) also differed significantly, with greater functional dependence and longer surgical delays observed in the delayed mobilization group. No significant differences were found in gender, fracture type or side, hypertension, osteoporosis, chronic obstructive pulmonary disease (COPD)/asthma, or smoking status (all p > 0.05).

**Table 2:** Comparison of baseline demographic and clinical characteristics between the early and delayed mobilization groups (N = 405). Note. Data are presented as n (%). COPD: Chronic obstructive pulmonary disease. Early mobilization was defined as mobilization within 48 hours after surgery (≤48 hours), whereas delayed mobilization was defined as mobilization more than 48 hours after surgery (>48 hours). The p values represent comparisons between the groups; p < 0.05 indicates statistical significance.

Variable	Early mobilization, ≤48 hours (n = 230)	Delayed mobilization, >48 hours (n = 175)	p-value
Age category, n (%)			
60-65 years	18 (7.8)	4 (2.3)	0.003
66-70 years	82 (35.7)	41 (23.4)	
71-75 years	106 (46.1)	80 (45.7)	
≥76 years	24 (10.4)	50 (28.6)	
Gender, n (%)			
Male	145 (63.0)	101 (57.7)	0.218
Female	85 (37.0)	74 (42.3)	
BMI category, n (%)			
<18.5 (underweight)	3 (1.3)	5 (2.9)	0.041
18.5-24.9 (normal weight)	82 (35.7)	45 (25.7)	
25.0-29.9 (overweight)	112 (48.7)	83 (47.4)	
≥30.0 (obese)	33 (14.3)	42 (24.0)	
Type of fracture, n (%)			
Femoral neck fracture	94 (40.9)	60 (34.3)	0.274
Intertrochanteric fracture	94 (40.9)	69 (39.4)	
Subtrochanteric fracture	42 (18.3)	46 (26.3)	
Side of fracture, n (%)			
Right	119 (51.7)	88 (50.3)	0.882
Left	106 (46.1)	82 (46.9)	
Bilateral	5 (2.2)	5 (2.9)	
Comorbidities, n (%)			
Hypertension	9 (3.9)	14 (8.0)	0.075
Diabetes mellitus	40 (17.4)	47 (26.9)	0.026
Coronary artery disease	58 (25.2)	64 (36.6)	0.013
Chronic kidney disease	48 (20.9)	57 (32.6)	0.008
Osteoporosis	30 (13.0)	29 (16.6)	0.338
COPD/asthma	3 (1.3)	6 (3.4)	0.151
Pre-fracture mobility status, n (%)			
Independent walking without an aid	70 (30.4)	24 (13.7)	<0.001
Occasionally uses a cane or walker	75 (32.6)	35 (20.0)	
Requires a wheelchair or full assistance	85 (37.0)	116 (66.3)	
Living situation, n (%)			
Living alone	78 (33.9)	39 (22.3)	0.002
With spouse/family	91 (39.6)	49 (28.0)	
Nursing home/assisted living	61 (26.5)	87 (49.7)	
Smoking status, n (%)			
Never smoked	84 (36.5)	54 (30.9)	0.194
Former smoker	111 (48.3)	87 (49.7)	
Current smoker	35 (15.2)	34 (19.4)	
Type of surgery, n (%)			
Hemiarthroplasty	16 (7.0)	21 (12.0)	0.021
Total hip replacement	125 (54.3)	77 (44.0)	
Internal fixation	89 (38.7)	77 (44.0)	
Time to surgery, n (%)			
<24 hours	60 (26.1)	21 (12.0)	<0.001
24-48 hours	79 (34.3)	35 (20.0)	
>48-72 hours	50 (21.7)	40 (22.9)	
>72 hours	41 (17.8)	79 (45.1)	
Assistive device use before fracture, n (%)			
None	47 (20.4)	16 (9.1)	<0.001
Cane	84 (36.5)	50 (28.6)	
Walker	61 (26.5)	33 (18.9)	
Wheelchair	38 (16.5)	76 (43.4)	

Table 3 shows the Spearman rank-order correlations among age, BMI, and total BI scores (N = 405). Age was significantly and negatively correlated with BMI (r = -0.182, p = 0.001) and total BI scores (r = -0.241, p = 0.001), indicating that increasing age was associated with lower BMI and reduced functional independence. Similarly, BMI showed a significant negative correlation with total BI scores (r = -0.296, p = 0.001), indicating that higher BMI was associated with lower functional status. Overall, all correlations were statistically significant and indicated negative relationships among the variables.

**Table 3:** Spearman’s rank-order correlation matrix for age, BMI, and total Barthel Index (BI) (N = 405). N: Total sample size; BI: Barthel Index. Negative correlation coefficients indicate inverse relationships between variables. **p < 0.01; ***p < 0.001. These symbols indicate statistical significance.

Variable	Age	BMI	Total Barthel Index (BI)
Age	-	rs​=-0.182***; p < 0.001	rs​=-0.241***; p < 0.001
BMI	-	-	rs​=-0.296***; p < 0.001
Total Barthel Index (BI)	-	-	-

Table 4 shows that patients in the early postoperative mobilization group (≤48 hours) had significantly higher mobility and functional independence scores than those in the delayed mobilization group (>48 hours). The early mobilization group had significantly higher mean ranks for baseline NMS (222.02 vs. 178.00; U = 15,750.00, Z = -4.05, p < 0.001), follow-up NMS (225.62 vs. 173.27; U = 14,923.00, Z = -4.50, p < 0.001), and total BI score (228.11 vs. 170.00; U = 14,350.00, Z = -5.00, p < 0.001). These results indicate that patients mobilized within 48 hours after surgery had significantly greater mobility and functional independence than those mobilized later.

**Table 4:** Comparison of mobility and functional independence between the early and delayed postoperative mobilization groups using the Mann–Whitney U test (N = 405). Note: Results are presented as mean ranks, sums of ranks, U statistics, Z statistics, and p values. N: Total sample size; BI: Barthel Index; NMS: New Mobility Score. Early mobilization was defined as mobilization within 48 hours after surgery (≤48 hours), whereas delayed mobilization was defined as mobilization more than 48 hours after surgery (>48 hours). ***p < 0.001 indicates statistical significance.

Variable	Mobilization timing after surgery	n	Mean rank	Sum of ranks	U	Z	p value
Baseline New Mobility Score (NMS)	≤48 hours (early)	230	222.02	51,065	15,750.00	-4.05	<0.001***
	>48 hours (delayed)	175	178	31,150	-	-	-
Follow-up New Mobility Score (NMS)	≤48 hours (early)	230	225.62	51,892	14,923.00	-4.5	<0.001***
	>48 hours (delayed)	175	173.27	30,323	-	-	-
Total Barthel Index (BI)	≤48 hours (early)	230	228.11	52,465	14,350.00	-5	<0.001***
	>48 hours (delayed)	175	170	29,750	-	-	-

Table 5 shows significant differences in mobility and functional independence between male and female patients with hip fractures. Male patients had higher mean ranks than female patients for baseline NMS (214.63 vs. 184.99; U = 16,696, Z = -4.50, p < 0.001), follow-up NMS (218.91 vs. 178.39; U = 15,644, Z = -5.00, p < 0.001), and total BI scores (221.37 vs. 174.58; U = 15,038, Z = -5.50, p < 0.001). These findings indicate that male patients had significantly higher mobility and functional independence scores than female patients.

**Table 5:** Comparison of mobility and functional independence between male and female patients after hip fracture surgery using the Mann-Whitney U test (N = 405). Note: Results are presented as mean ranks, sums of ranks, U statistics, Z statistics, and p values. N: Total sample size; BI: Barthel Index; NMS: New Mobility Score. ***p < 0.001 indicates statistical significance.

Variable	Gender	n	Mean rank	Sum of ranks	U	Z	p value
Baseline New Mobility Score (NMS)	Male	246	214.63	52,799	16,696.00	-4.5	<0.001***
	Female	159	184.99	29,416			
Follow-up New Mobility Score (NMS)	Male	246	218.91	53,851	15,644.00	-5	<0.001***
	Female	159	178.39	28,364			
Total Barthel Index (BI)	Male	246	221.37	54,457	15,038.00	-5.5	<0.001***
	Female	159	174.58	27,758			

Table 6 compares mobility and functional independence across age groups using the Kruskal-Wallis test (N = 405). For the baseline NMS, mean ranks progressively decreased with increasing age, from 237.14 in the 60-65-year age group (n = 22) to 175.27 among participants aged ≥76 years (n = 74), with a statistically significant overall difference (χ²(3) = 22.30, p < 0.001). A similar pattern was observed for the follow-up NMS, with mean ranks ranging from 236.18 in the 60-65-year age group to 173.18 in the ≥76-year age group (χ²(3) = 20.10, p < 0.001). For total BI scores, the highest mean rank was observed among participants aged 60-65 years (247.14), whereas the lowest was observed among those aged ≥76 years (168.26), with a statistically significant overall difference (χ²(3) = 25.40, p < 0.001). Overall, older age was associated with significantly lower mobility and functional independence scores.

**Table 6:** Comparison of mobility and functional independence across age groups using the Kruskal-Wallis test (N = 405). Note: Values are presented as mean ranks for each age group. The Kruskal-Wallis H test was used to assess overall differences across age categories. N: Total sample size; NMS: New Mobility Score; BI: Barthel Index; χ²: Chi-square statistic; df: Degrees of freedom. ***p < 0.001 indicates statistical significance.

Outcome	Age group	n	Mean rank	χ² (df = 3)	p value
Baseline New Mobility Score (NMS)	60-65 years	22	237.14	22.3	<0.001***
	66-70 years	123	216.52		
	71-75 years	186	201.05		
	≥76 years	74	175.27		
Follow-up New Mobility Score (NMS)	60-65 years	22	236.18	20.1	<0.001***
	66-70 years	123	220.41		
	71-75 years	186	199.42		
	≥76 years	74	173.18		
Total Barthel Index (BI)	60-65 years	22	247.14	25.4	<0.001***
	66-70 years	123	220.84		
	71-75 years	186	199.81		
	≥76 years	74	168.26		

**Figure 3:**
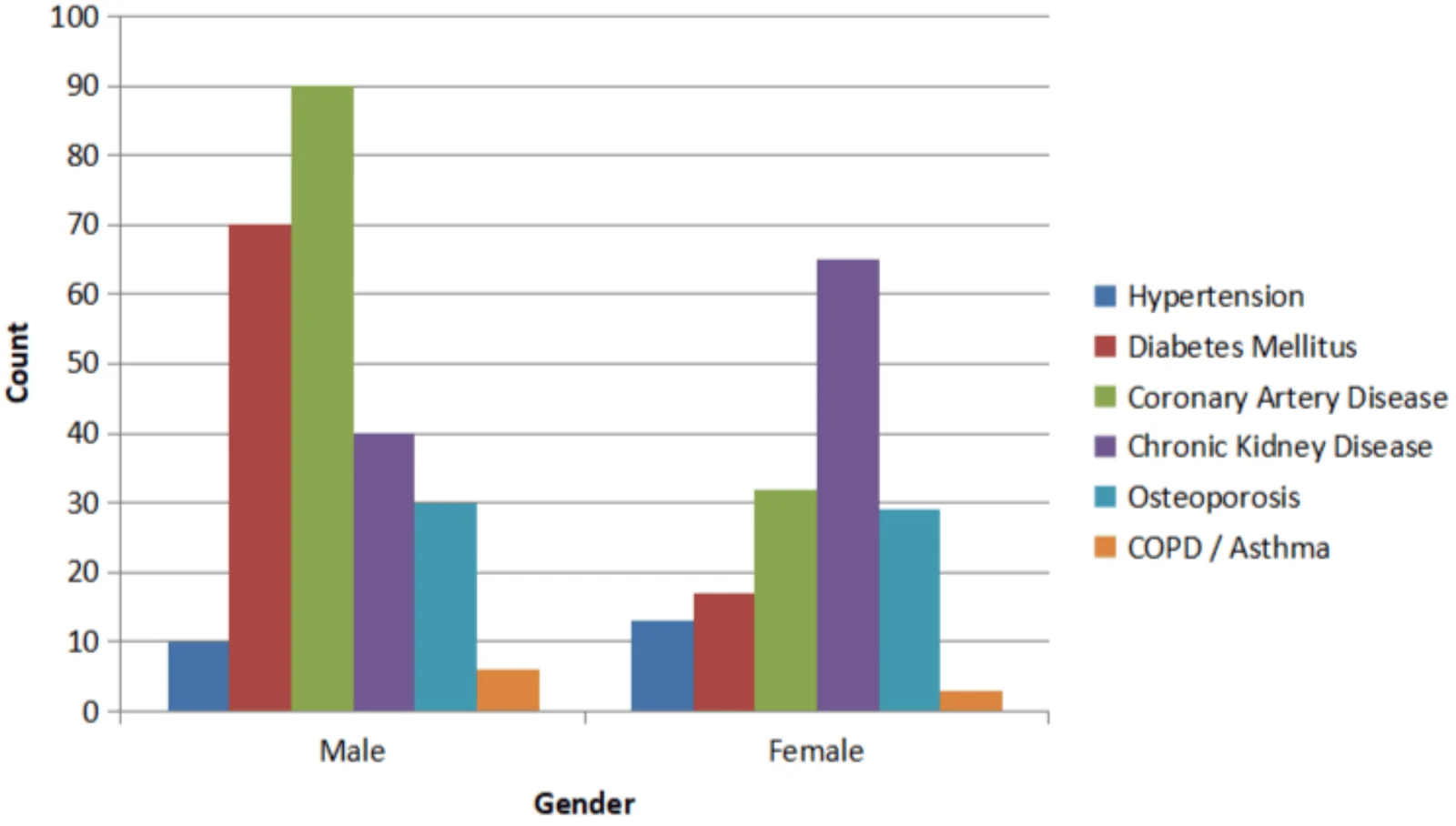
Distribution and total counts of comorbidities by gender among patients with hip fractures (N = 405).

Figure 3 illustrates the distribution of comorbidities among male and female patients who underwent hip fracture surgery. The most common comorbidities among male patients were coronary artery disease, diabetes mellitus, chronic kidney disease, and osteoporosis. Among female patients, the most common comorbidities were diabetes mellitus, coronary artery disease, osteoporosis, and chronic kidney disease. The prevalence of hypertension and other chronic conditions, such as COPD/asthma, was relatively low in both gender groups. Overall, the prevalence of coronary artery disease and diabetes mellitus was higher among male patients, whereas that of chronic kidney disease was higher among female patients.

Table 7 shows that delayed mobilization was significantly associated with lower odds of achieving a higher functional independence category at four weeks (OR = 0.54, 95% CI: 0.36-0.82, p = 0.003). Older age (OR = 0.96, 95% CI: 0.94-0.98, p = 0.001), higher BMI (OR = 0.75, 95% CI: 0.58-0.96, p = 0.025), greater comorbidity burden (OR = 0.58, 95% CI: 0.41-0.82, p = 0.002), poorer pre-fracture mobility (OR = 0.49, 95% CI: 0.35-0.69, p < 0.001), assistive device use (OR = 0.68, 95% CI: 0.50-0.93, p = 0.018), type of surgery (OR = 0.78, 95% CI: 0.62-0.98, p = 0.037), and longer time to surgery (OR = 0.98, 95% CI: 0.97-0.99, p = 0.003) were also independently associated with reduced functional independence. Gender was not significantly associated with functional outcomes (OR = 0.84, 95% CI: 0.63-1.12, p = 0.230). The proportional-odds assumption was satisfied (test of parallel lines, p = 0.684), and the model had a Nagelkerke R² value of approximately 0.39.

**Table 7:** Ordinal logistic regression analysis predicting four-week functional independence category based on mobilization timing and demographic, functional, and clinical factors (N = 405). Note. Nagelkerke R² = 0.39; Cox and Snell R² = 0.31; -2 log likelihood = 812.65. The test of parallel lines was not statistically significant (χ²(18) = 14.62, p = 0.688), supporting the proportional-odds assumption. The dependent variable was the four-week functional independence category derived from the Barthel Index (BI): 1 = total dependence (0-20), 2 = severe dependence (21-60), 3 = moderate dependence (61-90), and 4 = independence/minimal dependence (91-100). Ordinal logistic regression was performed using a proportional-odds model. Higher outcome categories indicate greater functional independence. Mobilization timing was coded as 1 = early mobilization (≤48 hours after surgery) and 2 = delayed mobilization (>48 hours after surgery). ORs <1 indicate reduced odds of achieving a higher functional independence category. VIF: Variance inflation factor; LL: Lower limit; UL: Upper limit. *p < 0.05; **p < 0.01; ***p < 0.001.

Predictor	B	SE	Wald χ²	p-value	OR	95% CI LL	95% CI UL	VIF
Mobilization timing (1 = early, ≤48 hours; 2 = delayed, >48 hours)	-0.62	0.21	8.72	0.003**	0.54	0.36	0.82	1.52
Age (years)	-0.04	0.01	10.56	0.001**	0.96	0.94	0.98	1.42
Gender (1 = male; 2 = female)	-0.18	0.15	1.44	0.23	0.84	0.63	1.12	1.18
BMI	-0.29	0.13	5	0.025*	0.75	0.58	0.96	1.46
Comorbidity burden	-0.55	0.18	9.33	0.002**	0.58	0.41	0.82	1.61
Pre-fracture mobility status	-0.71	0.17	17.39	<0.001***	0.49	0.35	0.69	1.7
Assistive device use before fracture	-0.38	0.16	5.64	0.018*	0.68	0.5	0.93	1.36
Type of surgery	-0.25	0.12	4.34	0.037*	0.78	0.62	0.98	1.28
Time to surgery (hours)	-0.018	0.006	9	0.003**	0.98	0.97	0.99	1.55

## Discussion

This study compared early and delayed postoperative mobilization in elderly patients undergoing hip fracture surgery and found that early mobilization (≤48 hours) was significantly associated with better functional outcomes at four weeks. In our study, age showed a weak but statistically significant negative correlation with BMI, suggesting that increasing age was associated with slightly lower BMI. This is consistent with a previous study showing that mean BMI decreased with age, indicating an age-related decline [18]. In our study, age was significantly negatively correlated with BI scores, indicating that increasing age was associated with reduced functional independence. This is consistent with evidence showing progressive age-related deterioration in the performance of ADLs among older people [19]. BMI also showed a significant negative correlation with BI scores, indicating that higher BMI was associated with reduced functional independence. This correlation was weak to moderate but aligns with previous research showing that low BMI was associated with higher ADL and instrumental activities of daily living (IADL) scores, supporting the negative association between BMI and functional status [20].

In our study, patients mobilized within 48 hours had significantly higher recalled baseline mobility scores than those mobilized later. Because baseline mobility preceded postoperative mobilization, this difference suggests possible selection bias rather than an effect of early mobilization. A large systematic review of patients with hip fractures found that early mobilization was associated with better subsequent mobility outcomes and improved performance in ADL [7]. The significant difference in baseline mobility between the early and delayed mobilization groups indicates potential selection bias and confounding by indication. Although we included pre-fracture mobility in the adjusted regression model, baseline adjustment cannot eliminate the possibility that patients with better pre-fracture functional capacity were more likely to receive early mobilization. Therefore, the observed association between early mobilization and better four-week outcomes should be interpreted cautiously and should not be considered causal. In our study, patients mobilized within the first 48 hours after surgery had significantly higher follow-up NMS than those mobilized later, indicating better short-term postoperative mobility. This is consistent with a cohort study of older adults who underwent hip fracture surgery, which found that early mobilization was significantly and independently associated with independent walking one year after surgery [21]. This finding is also consistent with a retrospective cohort study of elderly patients following hip fracture surgery, which showed that early mobilization was associated with significantly higher BI scores at six and 12 weeks after surgery, reflecting better functional recovery [22].

In our study, male patients had significantly higher baseline NMS than female patients. Previous research indicates that there may be sex-based disparities in hip fracture recovery, with women potentially recovering more slowly because of greater frailty and a higher burden of osteoporosis [23]. In our study, mean follow-up NMS were significantly higher among male patients than among female patients. This is consistent with previous studies indicating that male survivors continue to improve in functional measures such as gait speed and disability for up to six to 12 months after a hip fracture, whereas female survivors reach a plateau earlier, resulting in a persistent difference between the groups [24]. Our results also showed that male patients had significantly higher unadjusted BI scores than female patients, indicating greater functional capacity. However, other studies have shown that male sex is associated with worse functional outcomes after hip fracture, with men experiencing a more pronounced loss of function than women [23].

Mobility scores significantly declined with advancing age, with the lowest baseline and follow-up scores observed in the ≥76-year age group. This finding is consistent with previous studies indicating that older people are less likely to regain mobility after a fracture, demonstrating that age is an important predictor of functional recovery after hip fracture [25,26]. Similarly, functional independence, as indicated by the BI, decreased with age, with the oldest participants obtaining the lowest scores. This finding aligns with previous research indicating that recovery of ADL following hip fracture is poorer among elderly individuals, who are less likely to achieve full functional recovery in the long term [27].

Early mobilization remained independently associated with better functional independence at four weeks after adjustment for age, gender, BMI, comorbidities, and pre-fracture mobility [7]. Older age was associated with poorer functional independence [27], while male sex was associated with higher BI scores in the unadjusted analysis [23]. Higher BMI predicted reduced functional independence at four weeks, which may reflect obesity-related impairment in mobility and recovery; by contrast, very low BMI can indicate frailty or sarcopenia, both of which may negatively affect recovery outcomes [20].

Comorbidity burden was negatively associated with functional independence, and those with more health problems had lower BI scores at four weeks, which aligns with previous evidence on the influence of comorbid conditions such as diabetes, dementia, and hypertension on post-hip-fracture recovery [28]. Pre-fracture mobility also strongly predicted four-week functional outcomes, consistent with the literature, which found that lower baseline mobility is associated with a lower likelihood of regaining ADL performance and mobility after hip fracture [25]. Lastly, patients who used assistive devices before the fracture had lower levels of functional independence afterward, indicating slower early recovery, consistent with earlier studies showing that reliance on walking aids before hip fracture predicts poorer postoperative outcomes, including increased dependence and a greater likelihood of residential care [29].

Notably, almost half of the participants (49.6%) were wheelchair-dependent or required complete assistance before the fracture. The substantial proportion of participants who were wheelchair-dependent or required full assistance before the fracture also limits the uniform interpretation of postoperative “early mobilization,” because the clinical meaning and achievable level of mobility may differ considerably according to pre-fracture functional capacity. This was a baseline limitation that might have affected mobilization timing, as patients with worse functional status were less likely to be mobilized early. Thus, the observed association between early mobilization and better outcomes may partly reflect differences in baseline functional status rather than the effect of mobilization timing itself. This underscores the need to adjust for pre-fracture mobility when interpreting postoperative results.

The observed association should also be interpreted in the context of clinical heterogeneity within the cohort. Femoral neck, intertrochanteric, and subtrochanteric fractures may differ in their biomechanical characteristics, surgical management, weight-bearing restrictions, and rehabilitation trajectories. Similarly, bilateral fracture status and differences in postoperative pain, complications, and rehabilitation intensity may influence mobilization timing and subsequent functional recovery. These factors were not comprehensively captured in the present study and may have contributed to residual confounding. Future studies should incorporate more detailed fracture-specific, surgical, rehabilitation, and patient-level measures, including sarcopenia or muscle status, bone health, contralateral hip condition, and postoperative rehabilitation interventions, to better isolate the association between mobilization timing and functional recovery.

Overall, the findings suggest that early mobilization was associated with better short-term recovery and functional independence. However, because of the observational design and the potential for residual confounding, these findings should be interpreted as associations rather than evidence of causality. The results support consideration of early mobilization, when clinically appropriate, while accounting for individual patient stability and other clinical factors.

Limitations

Several limitations should be considered when interpreting these findings. First, the observational design limits causal inference. Although early mobilization was linked to better outcomes, the study cannot establish that early mobilization directly improved recovery, particularly given the baseline disparities between the groups that could have affected mobilization timing. Second, the study used convenience sampling, which may reduce generalizability. The sample was selected from a single hospital, and the recruited participants might not be representative of elderly patients with hip fractures in other geographic areas and healthcare settings. Third, baseline mobility was assessed using patients’ recall of pre-fracture function, which could introduce recall bias, especially among older adults. Patients might have over- or underestimated their pre-fracture function. Self-reported variables included pre-fracture mobility and assistive device use, which may have introduced recall bias and some variability between related variables. The high proportion of participants who were wheelchair-dependent or required full assistance before the fracture may also limit the uniform interpretation of postoperative mobilization, as the achievable level of functional recovery may differ according to pre-fracture functional capacity.

Fourth, follow-up outcomes were measured only four weeks after the operation. Recovery from a hip fracture is generally prolonged, and functional independence can continue to improve for months. Thus, longer-term outcomes, such as three- and six-month recovery outcomes, were not assessed. Postoperative analgesic protocols, regional nerve blocks, rehabilitation intensity, and standardized physiotherapy regimens were not systematically recorded and therefore could not be adjusted for in the analysis. Similarly, variations in surgical timing and procedure-specific rehabilitation protocols may have influenced postoperative mobilization and recovery. The cohort included different fracture types and surgical procedures, which may differ in their biomechanical characteristics, weight-bearing recommendations, and rehabilitation trajectories. Bilateral fracture cases may also have had different functional and rehabilitation trajectories from unilateral cases. Although these factors were considered where data were available, residual confounding due to clinical heterogeneity cannot be excluded.

Fifth, the study did not describe postoperative complications, such as delirium, infections, pressure ulcers, and deep vein thrombosis, in detail, which would have provided stronger clinical evidence regarding the effects of early mobilization. Lastly, although the regression analysis controlled for major factors, residual confounding may remain. Pain severity, physiotherapy intensity, surgical complexity, medication use, and hospital-based rehabilitation procedures were not measured in detail.

Future directions

Future longitudinal studies with prospective data collection should be conducted to better establish the temporal and causal relationship between mobilization timing and functional recovery. Future randomized controlled trials, where clinically and ethically feasible, may provide stronger evidence, while well-designed cohort studies may reduce selection bias. Longer follow-up periods (e.g., three, six, and 12 months) would help determine whether early mobilization has long-term benefits, such as greater independence, reduced institutionalization, and improved quality of life. Future research should also address complication-related outcomes, such as postoperative infections, thromboembolism, delirium, pressure ulcers, and hospital length of stay, as these are highly relevant to geriatric orthopedic care.

It would also be useful to investigate the impact of structured physiotherapy programs, including their frequency and intensity, on recovery. Rehabilitation success may depend not only on mobilization timing but also on standardized physiotherapy protocols. Lastly, subgroup analyses of high-risk patients, such as female patients, patients aged ≥76 years, patients with multiple comorbidities, and patients with poor pre-fracture mobility, should be conducted in future studies to develop specific rehabilitation approaches for vulnerable groups.

## Conclusions

Early mobilization within 48 hours after hip fracture surgery was associated with better short-term mobility and functional independence among older patients. Older age, greater comorbidity burden, poorer pre-fracture mobility, and pre-fracture assistive device use were also associated with less favorable functional outcomes. Given the observational design and potential for residual confounding, these findings should be interpreted as associations rather than evidence of causality. Nevertheless, the results support considering early mobilization when clinically appropriate, with the approach individualized according to each patient’s postoperative stability and overall clinical condition.
